# Utility of lung ultrasound in ANCA-associated vasculitis with lung involvement

**DOI:** 10.1371/journal.pone.0222189

**Published:** 2019-09-10

**Authors:** Natalia Buda, Anna Masiak, Zbigniew Zdrojewski

**Affiliations:** Department of Internal Medicine, Connective Tissue Diseases and Geriatrics, Medical University of Gdansk, Gdansk, Poland; Wayne State University, UNITED STATES

## Abstract

**Introduction:**

Granulomatosis with polyangiitis (GPA) and microscopic polyangiitis (MPA) are forms of antineutrophil cytoplasmic antibody (ANCA)-associated vasculitis (AAV). AAV most commonly affects the upper and lower respiratory tract as well as the kidneys. The first symptoms are often nonspecific, requiring careful differential diagnosis with infections and malignancies.

**Materials and methods:**

We analyzed the clinical and radiological data of 38 patients (20 females and 18 males) diagnosed with ANCA-associated vasculitis. Lung involvement was observed in 29 cases. Lung ultrasound (LUS) was performed on 21 patients from the study group and compared to chest CT. For 7 patients the examination was conducted repeatedly.

**Results:**

In total, 35 LUS and CT examinations were performed, revealing the following lesions: nodules, infiltrates with and without features of disintegration, caves (n = 17), diffuse alveolar hemorrhage (n = 3), and features of interstitial lung disease (ILD) with pulmonary fibrosis (PF) (n = 11). In 2 cases LUS and CT were negative. In 4 cases LUS was negative, despite a positive CT result.

**Conclusions:**

Both in CT and LUS, images of pulmonary lesions were consistent though highly variable. Therefore, further studies are required for a larger group of patients.

## Introduction

Granulomatosis with polyangiitis (GPA) and microscopic polyangiitis (MPA) are forms of antineutrophil cytoplasmic antibody (ANCA)-associated vasculitis (AAV). The inflammatory process damaging the vessel wall leads to impaired blood flow, ischemia and tissue necrosis. Additionally, GPA is associated with the development of extravascular necrotizing granulomas. The clinical presentation of AAV is very heterogeneous. The first symptoms are very often nonspecific and require careful differential diagnosis with infections and malignancies. AAV most commonly affects the upper and lower respiratory tract and kidneys. Diagnosis is based on the definition of the disease established during the conference in Chapel Hill in The United States of America in 1994 and on the criteria of classification developed in 1990. Nomenclature of the vasculitides was revised in 2012 by the American College of Rheumatology [1–3].

Vasculitis involving the airways is a common feature of AAV and can predate the diagnosis by years. Lung involvement is observed in 43% to 94% of patients with AAV [4–7]. In about 10% of cases, the lung is the only organ affected. The most common chest radiographic findings in GPA patients are solitary or multiple nodules and polymorphous infiltrates, usually located bilaterally, ranging in size from several millimeters to several centimeters. In about 25–50% of cases, infiltrates and nodules have a tendency towards becoming necrotic and consequently creating cavities [8–10]. Both in GPA and MPA features of diffuse alveolar hemorrhage (DAH) and pleural effusion may be detected [11]; enlarged hilar lymph nodes, pulmonary fibrosis and pleural lesions are less common [12,13]. It should be stressed that in as many as 30% of patients without clinical symptoms of lower respiratory tract involvement, abnormalities in chest imaging examinations can be found [9].

The efficacy of lung ultrasound (LUS) is very well documented in many pulmonary diseases, such as pneumonia, atelectasis, pulmonary edema, and pneumothorax [14–15]. Reports concerning LUS applicability for the assessment of pulmonary changes secondary to connective tissue disease focus primarily on fibrosis in interstitial lung disease (ILD). Single publications indicate its applicability also in diagnostics of other, less common complications secondary to systemic connective tissue disease, e.g., DAH [16–17].

The aim of this study was to assess lesions detected by ultrasound in patients with granulomatosis with polyangiitis (GPA) and microscopic polyangiitis (MPA) in comparison to abnormalities found by computed tomography (CT). To the best of our knowledge, this is the first comprehensive report devoted to this issue.

## Materials and methods

### Lung ultrasound (LUS) and computed tomography (CT)

LUS was performed in the sitting and lying positions, using the convex (2-6MHz) and linear (4-12MHz) transducers. The transducer was placed to each intercostal space over the chest wall (anterior, lateral and inferior) in the following lines: parasternal, middle clavicular, axillary (anterior, middle and posterior), scapular and paraspinal. Lesions detected in LUS and their locations were recorded in a dedicated form. The images obtained in LUS were compared to changes detected in CT scans. Chest CT was performed according to a standard protocol with the use of a 64-slice CT scanner made by GE. During the CT examination the patient was in the supine position. The CT scans were taken during a full inhalation, from the apex to the base of the lungs, with a section thicknesses of 2.5mm, continuously. Examinations were performed only after obtaining patients’ consent. The study protocol was approved by an independent local Bioethics Committee (Independent Bioethics Committee for Scientific Research at the Medical University of Gdansk NKBBN/474/2018).

### Statistical analysis

The statistical analysis was performed using Statistica 12 (StatSoft®, Tulsa OK). Descriptive statistics were used to show the characteristics of the study sample. Mean values were used with standard deviation (SD) in the case of quantitative variables and proportions in the case of categorical variables. Sensitivity, specificity, positive predictive values (PPV) and negative predictive values (NPV) were calculated.

## Results

### Characteristics of the study group

Between January 2013 and October 2017, 38 patients diagnosed with ANCA-associated vasculitis were treated in the Clinical Department of Internal Medicine, Connective Tissue Diseases and Geriatrics: 31 patients with granulomatosis with polyangiitis (GPA) and 7 patients with microscopic polyangiitis (MPA). The group comprised 20 females (18 diagnosed with GPA and 2 with MPA) and 18 males (13 with GPA and 5 with MPA), average age being 51.7 years (average age for females was 48.3 years, ranging from 26 to 69 years; average age for males was 55.6 years, ranging from 19 to 80 years). All patients presented the generalized form of disease. Anti-neutrophil cytoplasmic antibodies (ANCAs) were detected in 36 patients: proteinase 3 anti-neutrophil cytoplasmic antibodies (PR3-ANCAs) in 25 patients and myeloperoxidase anti-neutrophil cytoplasmic antibodies (MPO-ANCAs) in 11 patients; 2 female patients with dominant upper respiratory tract involvement were seronegative. Histopathological tests of affected organs were performed in 29 patients, including lung or bronchial biopsies in 10 patients. Vasculitis was confirmed in 10 patients. Lung involvement was detected in 29 patients. The dominant symptoms of the involved lower respiratory tract included: dyspnea (71%), cough (77%), and haemoptysis (17%). Table 1 presents systemic manifestations of vasculitis in analyzed group of patients.

**Table 1:** Systemic manifestations of ANCA-associated vasculitis in the analyzed group of 37 patients.

Affected systems and organs	GPA (31 patients)	MPA (7 patients)
Upper respiratory tract and facial skeleton	27	3
- sinuses	23	2
- nose	22	2
- eye/ orbital pseudotumor	15	1
- throat/larynx	7	1
- ear	13	0
- salivary glands	3	0
Lung	23	6
- infiltrates	21	5
- alveolar hemorrhage	4	1
Kidney	17	5
- kidney failure	12	3
- necessity of dialysis therapy due to acute kidney injury	1	1
Central nervous system	3	0
Peripheral nervous system	4	3
Heart	2	0
Arthritis and arthralgia	23	3
Gastrointestinal tract	3	0

### LUS and CT results

**Fig 1:**
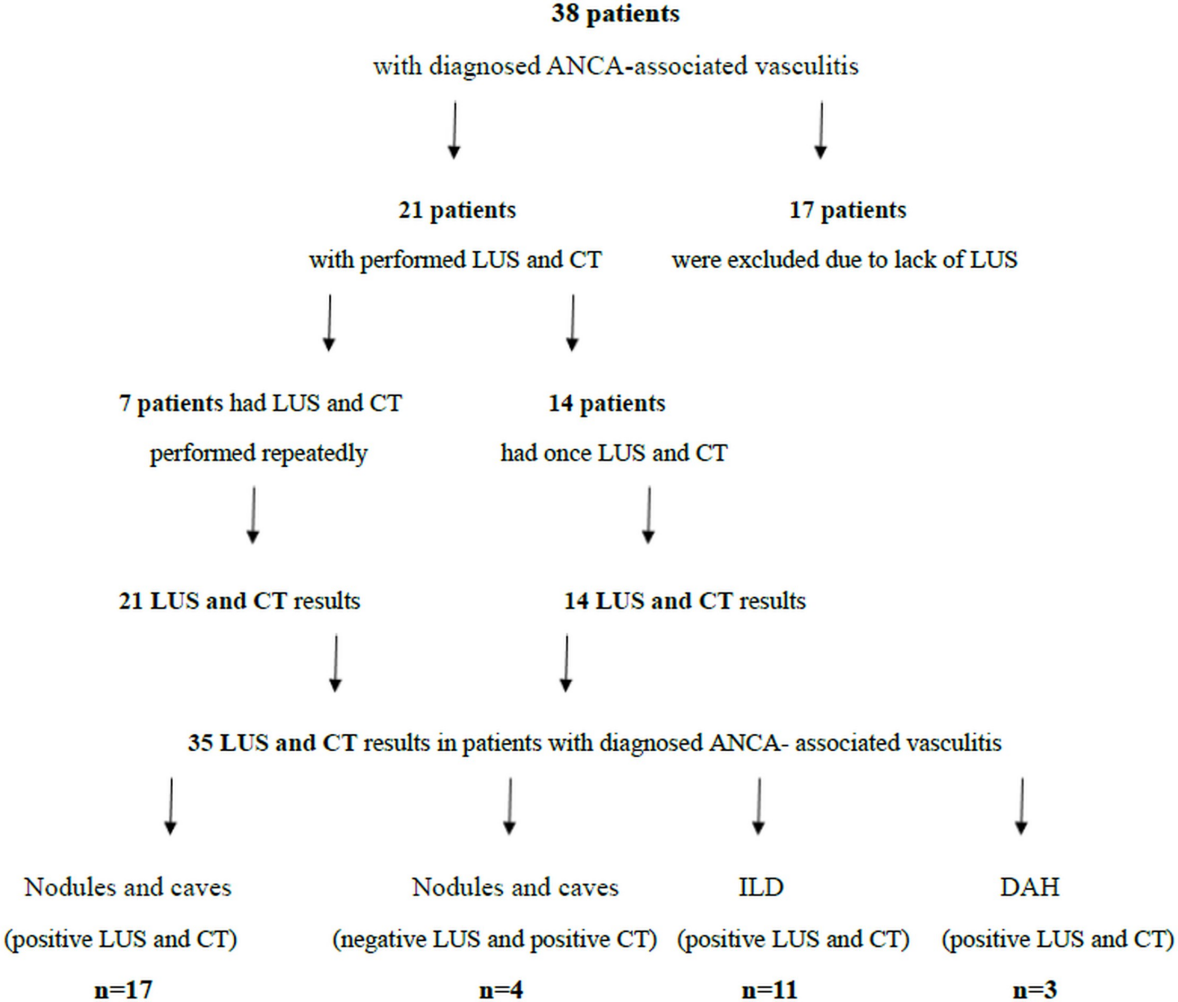
Flow chart of patients’ selection for the inclusion in the study and LUS results.

LUS and chest-CT were performed in 21 patients from the study population (Fig 1). In 7 patients the examinations were repeated. In total, 35 LUS and chest-CT examinations were performed: 26 during active vasculitis (disease activity according to the Birmingham Vasculitis Activity Score (BVAS) from 2 to 16 scores, average 5.9 scores); the remaining 9 examinations were performed during remission. Lung ultrasound and chest CT were not fully anonymized. Based on the conducted examinations, a comparative analysis of the results obtained with LUS and chest CT in patients diagnosed with systemic vasculitis and lung involvement was performed (Table 2).

**Table 2:** Collective results of imaging and clinical symptoms dominant in the group of patients. CT—computed tomography, LUS—lung ultrasound, pos.-positive, neg.—negative, No—number.

Lp	Patient No	Sex	Age (years)	Exam No	Symptoms			CT results			LUS results		
					dyspnea	cough	hemoptysis	cavity, nodules	ILD	DAH	cavity, nodules	ILD	DAH
1	1	F	35	1	**+**	**+**	**-**	**+**	**-**	**-**	**+**	**-**	**-**
2			35	2	**+**	**+**	**+**	**+**	**-**	**-**	**+**	**-**	**-**
3			36	3	**+**	**+**	**-**	**+**	**-**	**-**	**+**	**-**	**-**
4			38	4	**+**	**+**	**-**	**+**	**-**	**-**	**+**	**-**	**-**
5	2	M	80	1	**+**	**+**	**+**	**+**	**+**	**-**	**+**	**+**	**-**
6			80	2	**+**	**+**	**-**	**+**	**-**	**-**	**+**	**-**	**-**
7			81	3	**+**	**+**	**-**	**-**	**-**	**+**	**-**	**-**	**+**
8			81	4	**+**	**+**	**-**	**-**	**-**	**+**	**-**	**-**	**+**
9	3	F	61	1	**+**	**+**	**-**	**+**	**-**	**-**	**+**	**-**	**-**
10			61	2	**+**	**+**	**-**	**+**	**-**	**-**	**+**	**-**	**-**
11	4	F	58	1	**-**	**-**	**-**	**+**	**-**	**-**	**+**	**-**	**-**
12			58	2	**+**	**+**	**-**	**+**	**-**	**-**	**+**	**-**	**-**
13	5	M	27	1	**-**	**-**	**+**	**+**	**-**	**-**	**+**	**-**	**-**
14			28	1	**-**	**-**	**-**	**+**	**-**	**-**	**+**	**-**	**-**
15			27	3	**-**	**-**	**-**	**+**	**-**	**-**	**+**	**-**	**-**
16	6	M	52	1	**+**	**+**	**-**	**+**	**-**	**-**	**+**	**-**	**-**
17	7	M	32	1	**-**	**-**	**+**	**+**	**-**	**-**	**-**	**-**	**-**
18	8	F	63	1	**+**	**+**	**-**	**-**	**+**	**+**	**-**	**+**	**+**
19	9	M	48	1	**+**	**+**	**-**	**-**	**+**	**-**	**-**	**+**	**-**
20			48	2	**+**	**+**	**-**	**-**	**+**	**-**	**-**	**+**	**-**
21	10	M	28	1	**-**	**-**	**-**	**+**	-	**-**	**-**	**-**	**-**
22	11	M	62	1	**-**	**-**	**-**	**-**	**+**	**-**	**-**	**+**	**-**
23	12	M	56	1	**+**	**+**	**+**	**+**	**-**	**-**	**+**	**-**	**-**
24	13	M	64	1	**+**	**+**	**+**	**-**	**+**	**-**	**-**	**+**	**-**
25	14	M	63	1	**+**	**+**	**-**	**-**	**+**	**-**	**-**	**+**	**-**
26	15	M	51	1	**+**	**-**	**-**	**+**	**-**	**-**	**-**	**-**	**-**
27	16	M	65	1	**+**	**+**	**-**	**-**	**+**	**-**	**-**	**+**	**-**
28			66	2	**+**	**+**	**-**	**-**	**+**	**-**	**-**	**+**	**-**
29	17	M	46	1	**+**	**+**	**-**	**+**	**-**	**-**	**+**	**-**	**-**
30	18	M	61	1	**-**	**+**	**-**	**+**	**-**	**-**	**-**	**-**	**-**
31	19	M	51	1	**+**	**+**	**-**	**-**	**+**	**-**	**-**	**+**	**-**
32			51	2	**+**	**+**	**-**	**-**	**+**	**-**	**-**	**+**	**-**
33	20	F	50	1	**+**	**+**	**-**	**+**	**-**	**-**	**+**	**-**	**-**
34	21	F	30	1	**-**	**+**	**-**	**-**	**-**	**-**	**-**	**-**	**-**
35			33	2	**-**	**+**	**-**	**-**	**-**	**-**	**-**	**-**	**-**

**Fig 2:**
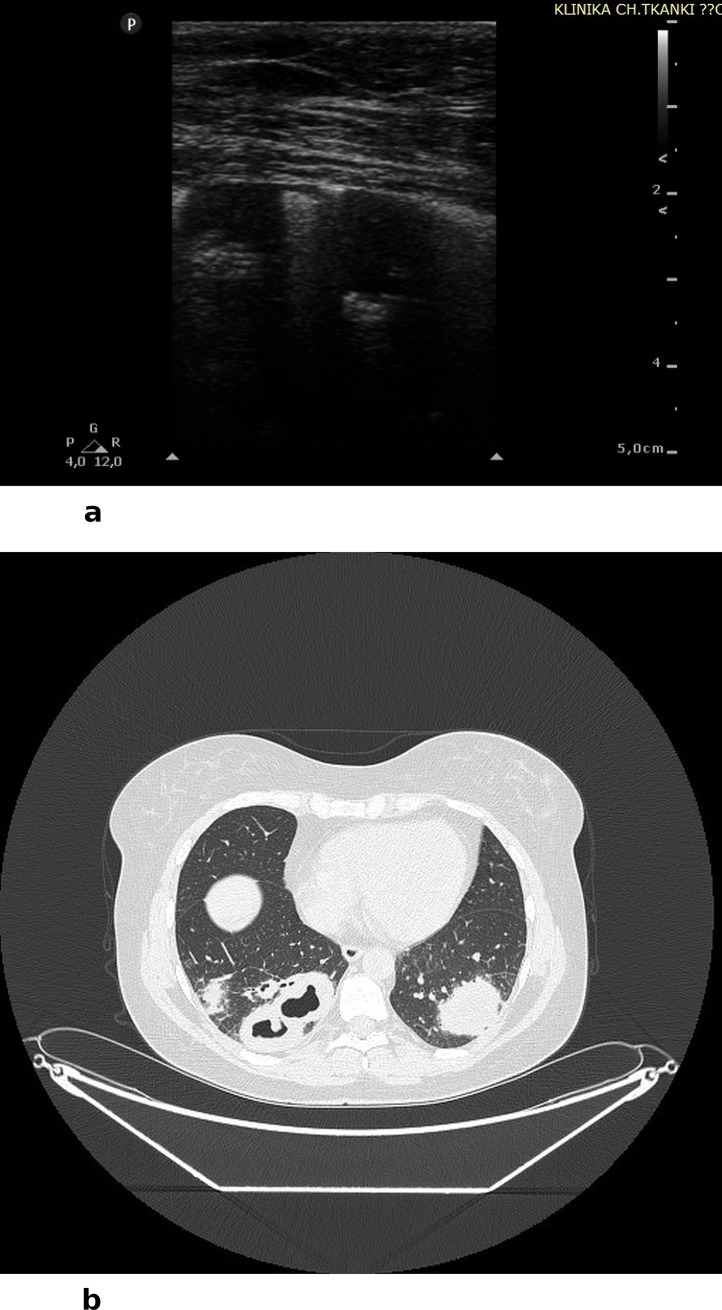
A) LUS: two round hypoechoic consolidations (infiltrates), partly disintegrated, hypoechoic pulmonary pleura, B) CT: Subpleural round lesions with features of disintegration, creating caves.

**Fig 3:**
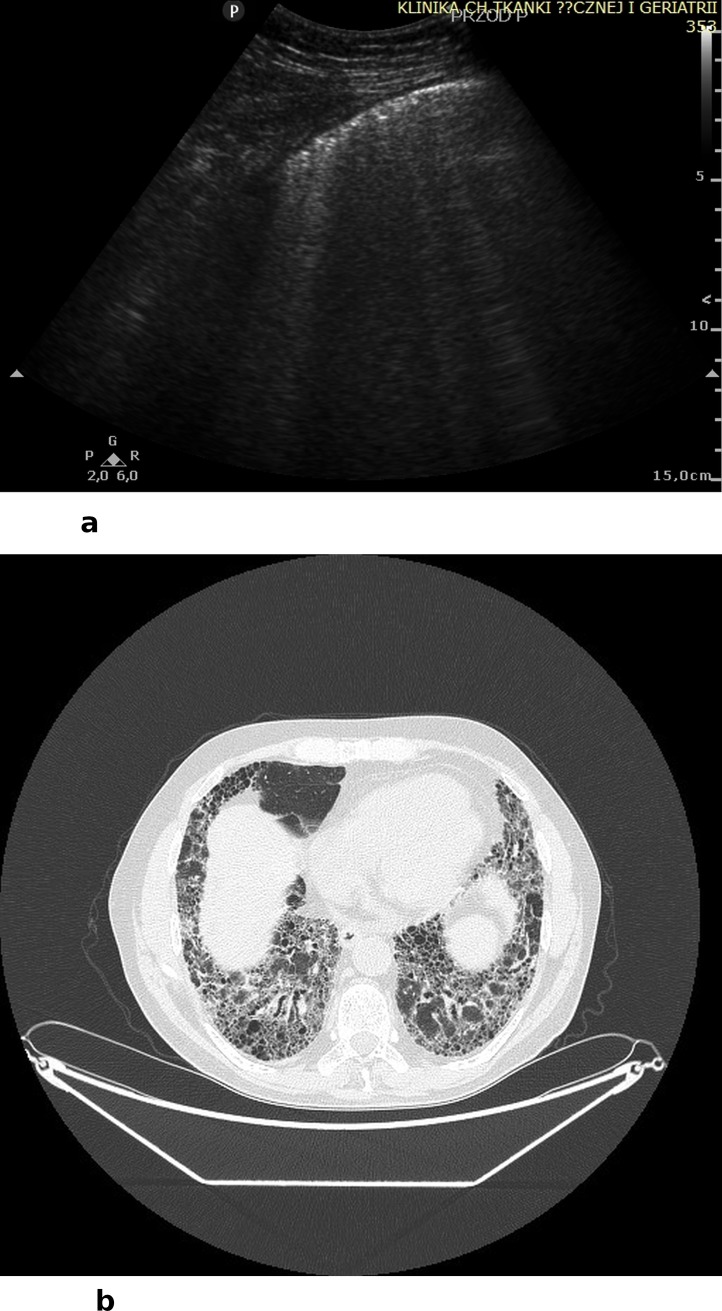
A) LUS: Irregular pleural line, fragmentarily visualized, with B-line artifacts; ILD secondary to MPO-ANCA-positive vasculitis, B) CT: Bilateral honeycombing, features of pulmonary fibrosis secondary to MPO-ANCA-positive vasculitis.

**Fig 4:**
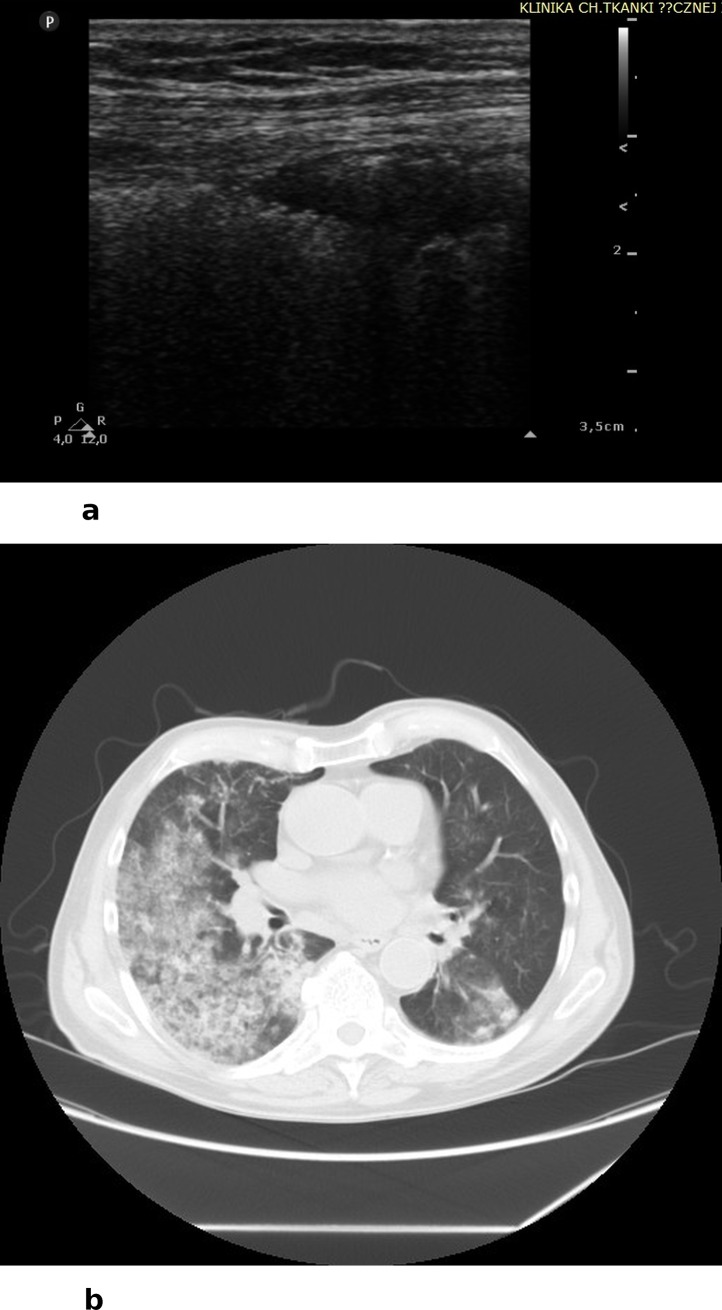
A) LUS: oval, hypoechoic subpleural consolidation in DAH, B) CT: Features of alveolar hemorrhage secondary to vasculitis.

In 17 cases, LUS revealed infiltrates as well as infiltrates with features of disintegration and cavities (Fig 2A). The visualized infiltrates and caves include only these lesions that were adjacent to the line of pleura. Subpleural infiltrates in ultrasound are visualized as hypoechoic round or oval consolidations, without central flow visible in color Doppler (CD) and power Doppler (PD) modes. Caves visualized in LUS were round and anechoic; flow in CD and PD modalities was also absent. In some cases we observed hypoechoic round or oval infiltrates with features of disintegration, partly filled in with fluid content (anechoic). In 11 cases features of interstitial lung disease (ILD) were detected, with bilateral B-line artifacts and co-occurring lesions within the pleural line visible in LUS (Fig 3A). B line artifacts were present > 3 in one intercostal space and they were secondary to the presence of pleural changes. Changes in the pleural lines included irregularity, fragmentation, boldness and blur. However, it is important to hightlight, that the described interstitial lesions in LUS may be present in any other form of ILD (not associated with vasculitis). In 3 cases, LUS visualized features of diffuse alveolar hemorrhage (DAH) secondary to infectious pulmonary vasculitis. Features of hemorrhage were visualized in LUS as bilateral but uneven spaced B-line artifacts (> than 3 in one intercostal space) and hypoechoic consolidations of homogenous textures, shaped as pulmonary lobules, with partly stopped flow in vessels of the subpleural region and pleural effusion (Fig 4A). Both in patients with interstitial lesions and alveolar hemorrhage, attention must be paid to the technique of performing LUS. In both cases B-line artifacts are present, which are well visualized with the convex transducer. However, due to a variety of reasons for the presence of this type of artifact in the LUS image, it is of a low diagnostic specificity. Thus, it was necessary to use a linear transducer and visualize lesions within the line of pleura and in the subpleural region. Coexistence of ILD and lung cavities occurred in 1 patient, and ILD with DAH also occurred in 1 patient. In 4 cases, LUS did not reveal any lesions, despite AAV diagnosis, and pulmonary lesions confirmed with CT. Two studies (LUS and CT), despite earlier involvement of the respiratory system, were completely negative. These tests were performed during remission of the underlying disease. The sensitivity of LUS as compared to computed tomography (CT) in diagnosing lesions secondary to GPA amounted 89,7%. However, the specificity as well as PPV and NPV are unreliable due to the lack of anonymisation of the study and the evaluation of only patients with a recognized GPA.

Chest CT was performed at the diagnostic stage and then again during treatment to assess disease activity. The majority of patients in the study group (n 12) manifested infiltrations with features of disintegration and caves (Fig 2B), in 8 patients lesions typical of ILD were detected (Fig 3B), and in 2 cases features of DAH (Fig 4B). It should be stressed that, considering the length of the observation period, the average number of examinations performed for one patient was: CT– 5.52 times, chest X-ray 2.57 times, respectively.

## Discussion

In this study we analyzed the clinical data of patients diagnosed with ANCA-associated vasculitis with lung involvement. Both in CT and LUS, images of pulmonary lesions were highly variable. The detected lesions included nodules, infiltrations with and without features of disintegration, caves, alveolar hemorrhage, and features of ILD with pulmonary fibrosis. This diversity of radiological images is typical of ANCA-associated vasculitis [1,2]. Irrespective of the type of pulmonary vasculitis, patients require long-term care and monitoring since this disease has phases of exacerbations and remissions. Additionally, due to the administered immunosuppressive therapy, patients with AAV are at risk of infectious complications, also involving the respiratory tract [6–9]. This clinical condition necessitates repeated imaging examinations thus exposing the patient to a cumulative dose of ionizing radiation [18–19]. The average number of X-ray and CT examinations performed on the analyzed patients amounted to 2.57 and 5.52 per patient, respectively. Considering that the risk for developing cancer in patients with GPA is 2-fold higher than in the general population [18], the possibility of limiting exposure to ionizing radiation through the application of LUS as the diagnostic modality appears extremely inviting. Due to lack of ionizing radiation, LUS can be repeatedly performed. In addition, ultrasound examination can be performed during hospitalization at the patient's bedside as well as during a visit to the rheumatologist's office. The aforementioned diversity of pulmonary lesions implicates some limitations of LUS as compared to CT. The basic limitation of this modality of lung imaging is the absence of contact between the lesion and the pulmonary pleura [20,21]. In our study, the most of the patients manifested diffuse lesions, which definitely facilitated their detection by an ultrasound operator. Recognizing this limitation, the disease course and treatment effectiveness can be monitored based on repeatedly performed assessment. In the analyzed group, only in 4 cases, LUS did not detect pathology although lesions were visualized in chest CT. In the first case, lesions did not directly involve the pulmonary pleura, despite the active phase of the disease. In three other cases the inflammatory process was in the remission phase, and residual lesions did not directly involve the pulmonary pleura. In the remaining cases the images obtained in LUS were consistent with the results of chest CT. The sensitivity of LUS as compared to chest CT in the study group was 79%. These results are very promising. However, further studies conducted on larger and more uniform groups of patients are necessary to verify the efficacy of LUS in the diagnosing and monitoring of vasculitis with pulmonary involvement.

## Conclusions

In most of the cases LUS yields results consistent with CT, despite a variety of pulmonary lesions secondary to vasculitis. The major limitations are infiltrations, with features of disintegration that do not involve directly the line of pleura. In order to assess the sensitivity and specificity of LUS in relation to chest CT, further studies should be conducted on a larger patient group.
